# Microsatellite loci cross-species transferability in *Aedes fluviatilis* (Diptera:Culicidae): a cost-effective approach for population genetics studies

**DOI:** 10.1186/s13071-015-1256-9

**Published:** 2015-12-15

**Authors:** Laura Cristina Multini, Mauro Toledo Marrelli, André Barretto Bruno Wilke

**Affiliations:** Institute of Tropical Medicine of São Paulo, University of São Paulo, Av. Dr. Enéas de Carvalho Aguiar, 470, São Paulo, SP CEP 05403-000 Brazil; Department of Epidemiology, School of Public Health, University of São Paulo, Av. Dr. Arnaldo 715, São Paulo, SP CEP-01246-904 Brazil

**Keywords:** *Aedes fluviatilis*, Microsatellite, Culicidae, Genetic structure

## Abstract

**Background:**

*Aedes fluviatilis* is a neotropical mosquito species thought to be a potential vector of Yellow Fever viruses and can be infected with *Plasmodium gallinaceum* in laboratory. A better understanding of its genetic structure is very important to understand its epidemiologic potential and how it is responding to urbanization. The objective of this study was to survey the transferability of microsatellites loci developed for other *Aedes* to *Ae. fluviatilis.*

**Findings:**

We tested in *Ae. fluviatilis* 40 pairs of primers known to flank microsatellite regions in *Aedes aegypti, Aedes albopictus* and *Aedes caspius*, and found eight loci that amplified consistently. The number of alleles per locus ranged from 2 to 15, and the expected heterozygosity ranged from 0.09 to 0.85.

**Conclusions:**

We found that several microsatellite primers successfully transferred to *Ae. fluviatilis*. This finding opens avenues for cost-effective optimization of high-resolution population genetic tools.

## Findings

### Background

*Aedes fluviatilis* (Lutz, 1904) [1] is a neotropical species found in Central and South America [2]. These mosquitoes can be found in wild, semi wild, urbans and sub-urbans environments [3]. Females are highly anthropophilic, more active during the day but can also bite at night and have been observed blood feeding while already developing eggs [4]. *Ae. fluviatilis* mosquitoes are considered a potential vector of the yellow fever viruses, are naturally infected with *Wolbachia* (w*Flu*) and can be experimentally infected with *Plasmodium gallinaceum* [5–7].

Previous studies showed that *Ae. fluviatilis* is adapted to the urban environment [8, 9]. Considering, the scarce information about this species biology and ecology, a better understanding of its genetic structure is important to access its epidemiologic potential and how it is responding to increasing urbanization. However, no molecular markers are currently available for population genetic studies to better understand adaptation and selection processes affecting this culicid.

Microsatellites or Simple Sequence Repeats (SSR) are molecular markers commonly used for population genetics studies, they are repetitive non-codon DNA regions composed by 1 to 6 base pairs in tandem, present in eukaryotic and prokaryotic genomes and are very useful to analyze the genetic structure of mosquitoes [10, 11]. However only a few species have specific designed microsatellite primers and their development is not a simple task, a fact that limits its utilization in a broader spectrum of species.

Transferability of microsatellite loci between close taxa have been successfully performed before [12, 13], reducing primer development cost encouraging studies of population genetic structure in species with no specific developed microsatellite loci. Our objective was to survey the transferability to *Ae. fluviatilis* of microsatellite primers previously developed for *Aedes aegypti*, *Aedes albopictus* and *Aedes caspius.*

### Methods

Forty microsatellite primers originally designed for *Ae. aegypti*, *Ae albopictus* or *Ae. caspius* were tested in nine *Ae. fluviatilis’* populations composed of 30 female mosquitoes each, comprising a total of 270 individuals, collected in urban parks throughout the city of São Paulo [8]. DNA samples were extracted using the DNeasy Blood and Tissue Kit (Qiagen, Hilden, Germany), following the manufacturers’ protocol.

PCR reactions were performed as in Huber et al. [14], Porretta et al. [15], Porretta et al. [16], Chambers et al. [17], Delatte et al. [18], Beebe et al. [19] in an E6331000025 Eppendorf Thermocycler (Masterclycler Nexus Gradient, Eppendorf, Hamburg, Germany). A gradient PCR was performed to identify the ideal annealing temperature for primers that did not amplify with the developer’s original protocol by testing identical PCR reactions across a range of annealing temperatures. Successful amplifications were size sorted in 1 % agarose gels, stained with GelRed^™^ dye (Biotium, Hayward, CA, USA) and examined under UV light.

After incorporation of the fluorescent dye (FAM, HEX and NED), PCR products were diluted (1:7) by mixing 3 μL of each product with 21 μL of ultra-pure water for a final volume of 30 μL. A second dilution was performed with 2 μL of the previous dilution suspended in 8.925 μL of formamide HIDI (Applied Biosystems, Foster City, CA, USA) and 0.075 μL of molecular weight marker GeneScan 500 ROX (Applied Biosystems, Foster City, CA, USA) for a final volume of 11 μL. The samples were sent to the Centro de Estudos do Genoma Humano da Universidade de São Paulo and processed in the automatic sequencer ABI 3730 (Applied Biosystems, Foster City, CA, USA). Fragment analyzes were performed on the software Gene Marker (v1.85 SoftGenetics, Centre County, State College, PA, USA). The allele number, observed heterozygosity (*H*_*O*_), expected heterozygosity (*H*_*E*_), deviations from Hardy-Weinberg equilibrium and assessment of linkage disequilibrium were calculated in Genepop (v4.2 http://genepop.curtin.edu.au/) [20] and Arlequin (v3.5) [21].

### Results and discussion

Our results show that several primers were successfully transferred to *Ae. fluviatilis*, which corroborates the results of Bello & Becerra [12] that successfully used microsatellite primers developed for *Ae. caspius* in *Ae. taeniorhynchus*. The 8 microsatellite primer pairs that successfully transferred to *Ae. fluviatilis* (OchcB5, OchcB9, OchcD11, AEDC, Albtri-3, Albtri-20 Albtri-33 and Albtri-44) (Table 1) were found to be moderately polymorphic, which may not be surprising since microsatellite loci in *Aedes* mosquitoes are often less polymorphic than loci developed for other Culicidae genera (e.g. *Culex quinquefasciatus* and *Anopheles funestus*) [22–24].

**Table 1:** Microsatellite loci amplified in *Aedes fluviatilis*

Locus	Sequences 5′-3′	Repetitive motif	Source species	T (°C)	Size range	References
OchcB5	F: GCTTCGAATCTTGATGAGCA	CAC	*Ae. caspius*	52* (55)	194-196* (155–157)	Porretta et al. [15]
	R: TGGATGCAGAGTGTT TTGGA					
OchcB9	F: CCAAAACACTCTGCATCCAA	CTA	*Ae. caspius*	55	90-288* (272–282)	Porretta et al. [15]
	R: GGACTGGCCGAATACAGAGA					
OchcD11	F: TTCGACTCAGTTCGACGAGA	GT	*Ae. caspius*	55	88-132* (135–148)	Porretta et al. [15]
	R: GGTCAATTCGGTTGAGTGGTT					
Alb-tri-3	F: AGATGTGTCGCAATGCTTCC	AGA	*Ae. albopictus*	56	93-453* (123–153)	Beebe et al. [19]
	R: GATTCGGTGATGTTGAGGCC					
Alb-tri-20	F: GTGCCGTTGATCATCCTGTC	GTG	*Ae. albopictus*	56	105-120* (165–201)	Beebe et al. [19]
	R: TCCAGCACCGTGAGTAATCC					
Alb-tri-33	F: GGCTGCTGTTGTTGGTACG	GGC	*Ae. albopictus*	56	102-219* (137–182)	Beebe et al. [19]
	R: CACGTTCAATCACCGGTTCC					
Alb-tri-44	F: CACTCGCGCGTGTTCTTC	CAC	*Ae. albopictus*	56	165-180* (173–212)	Beebe et al. [19]
	R: GACGCACCATCAGCATCATC					
AEDC	F: TGCAGGCCCAGATGCACAGCC	GTA	*Ae. aegypti*	60	246-477* (210–230)	Chambers et al. [17]
	R: TCCGCTGCCGTTGGCGTGAAC					

T = annealing temperature; *Values found in *Aedes fluviatilis*. In parenthesis, size range found in original studies

The number of alleles ranged from 2 to 15 per locus per population, the allele sizes were different from the alleles found in original manuscripts, except for locus OchcB5. Although locus Albtri3 had a high number of alleles per locus in *Ae. fluviatilis*, two alleles were found most frequently in the populations examined. Tests for Hardy-Weinberg equilibrium were made for the 8 functional microsatellite loci (Table 2). The *H*_*E*_ values were higher than the *H*_*O*_ values in 5 of 8 conducted tests (Table 2). After 150 possible tests, linkage disequilibrium was found between the loci OchcB9 and Albtri20; OchcB9 and Albtri33; OchcD11 and Albtri3; Albtri33 and Albtri20; and OchcD11 and Albtri33, however none of the linkages were considered significant, once no two loci were consistently correlated across the tested mosquitoes. The highest level of polymorphism was found in locus Albtri3. In contrast OchcB5 was close to monomorphic and might not be useful for *Ae. fluviatilis* genetic structure studies although additional populations should be tested. Both Albtri20 and Albtri33 were monomorphic in one of the nine populations. The other loci were moderately polymorphic and potentially very useful in future population genetics analyses of this species.

**Table 2:** Characterization of microsatellite loci in *Aedes fluviatilis*

Locus	N_a_	*H*_*O*_	*H*_*E*_	*P*
OchcB5	2	0.00000	0.09248	**0.00000**
OchcB9	5	0.30224	0.37172	**0.00000**
OchcD11	4	0.92164	0.56264	**0.00000**
Albtri3	15	0.68605	0.85359	0.01953
Albtri33	3	0.13704	0.14910	0.00495
Albtri20	6	0.11236	0.11912	0.01130
AEDC	3	0.95880	0.50203	**0.00000**
Albtri44	11	0.89057	0.69567	**0.00000**

All 270 individuals were analyzed with 8 microsatellite loci; Na = Number of alleles found in each locus; *H*_*O*_ = Observed Heterozygosity; *H*_*E*_ = Expected Heterozygosity; *P* value of Hardy-Weinberg equilibrium, in bold significant values after Bonferroni correction

Null alleles, which occur when mutations in the primer regions prevent primers from annealing, are commonly found in studies using microsatellite markers [23]. Their presence can lead to incorrect assumptions of low genetic diversity and possibly consequent overestimation of Wright’s *F*-statistics values [25]. Although caution is recommended regarding the use of microsatellite loci that may have null alleles, as they are less informative and may be more ambiguous, they still could be included in the analysis if their bias do not influence the results significantly. [13, 23, 25]. Also, cross-species microsatellite loci transfer might result in an ascertainment bias, which means that rates of variability may be consistently lower in the new species than in the species for which the markers were developed. This may be erroneously interpreted as evolutionary dynamics fluctuations among species. However, this bias is drastically diminished when the target and original populations have distinct demographic patterns [26].

Cross-species microsatellite loci transferability from *Ae. aegypti*, *Ae. albopictus* and *Ae. caspius* to *Ae. fluviatilis* demonstrate that loci developed for a few species can be recast for population genetic studies of less well studied species. This finding may open a new range of genetic structure studies of poorly known and/or neglected mosquitoes, e.g. secondary epidemiological importance species as well as a cost-effective genetic population accessible tool.
